# Perceived barriers to healthcare for persons living in poverty in Quebec, Canada: the EQUIhealThY project

**DOI:** 10.1186/s12939-015-0135-5

**Published:** 2015-01-17

**Authors:** Christine Loignon, Catherine Hudon, Émilie Goulet, Sophie Boyer, Marianne De Laat, Nathalie Fournier, Cristina Grabovschi, Paula Bush

**Affiliations:** Department of Family Medicine, Faculty of Medicine and Health Sciences, University of Sherbrooke, Quebec, Canada; ATD Fourth World Movement Canada, Quebec, Canada; Academic Primary Care Unit, Charles-LeMoyne Hospital, Quebec, Canada; Participatory Research at McGill (PRAM), McGill University, Quebec, Canada

**Keywords:** Primary healthcare, Equity, Poverty, Quality of care, Participatory research, Social assistance

## Abstract

**Introduction:**

Ensuring access to timely and appropriate primary healthcare for deprived patients is an issue facing all countries, even those with universal healthcare systems. There is a paucity of information on how patients living in a context of material and social deprivation perceive barriers in the healthcare system. This study combines the perspectives of persons living in poverty and of healthcare providers to explore barriers to responsive care for underserved persons with a view to developing equity-focused primary care.

**Methods:**

In this participatory action research we used photovoice, together with a method known as ‘merging of knowledge and practice’ developed by ATD Fourth World, an international community organization working to eradicate poverty. The study was conducted in two teaching primary care practices in the Canadian province of Quebec. Participants consisted of 15 health professionals and six members of ATD Fourth World; approximately 60 group meetings were held. Data were analyzed through thematic analysis, in part with the involvement of persons living in poverty.

**Results:**

Three main barriers to responsive care in a context of poverty were highlighted by all participants: the difficult living conditions of people living in poverty, the poor quality of interactions between providers and underserved patients, and the complexity of healthcare system organization and functioning.

**Conclusion:**

Our research revealed that unhealthy living conditions prevent persons living in poverty from accessing quality healthcare and maintaining good health. Also, the complexity of the healthcare system’s organization and functioning has a negative impact on the interactions with healthcare providers. Changes in policy and practice are needed to address those barriers and to achieve greater equity and provide more responsive care for persons living in poverty.

## Introduction

Healthcare systems can increase health inequalities if they do not take into account the needs and socioeconomic living conditions of underserved populations [1,2]. The most recent Marmot report [3] draws attention to the alarmingly low consideration given to the causes of social inequalities in the English healthcare system. In Canada and Quebec, barriers to universal healthcare represent a heavy burden for certain populations, particularly people living on a low income or social assistance, people with disabilities, Aboriginal peoples, and recent immigrants and refugees in precarious situations [4-6]. Persons living in poverty (PLPs) are at greater risk for deterioration in health status, chronic illnesses, and premature death than are affluent persons [5,7-11]. Yet there is a growing body of evidence indicating that PLPs receive the least amount of healthcare (known as the inverse care law) [12,13]. PLPs are less likely to have a family physician and to obtain preventive and secondary care, and more likely to report negative experiences of care [4-6,14-19].

Compared to moderate and high-income populations, PLPs experience greater need for support to access preventive care and improved care experiences. Yet adequately responding to these needs is often challenging for front-line healthcare providers (HCPs), specifically family physicians and primary care teams [20-23]. A deeper understanding of barriers to healthcare access, as well as of their causes and impacts on PLPs’ experience of care would help HCPs overcome these challenges and provide socially responsive care. Findings from the few such studies suggest that the reasons for PLPs’ avoidance or cessation of use of various health services include a perception that HCPs do not listen to them or are rude, judgmental, or controlling [24,25]. PLPs also believe their poverty affects the quality of care they receive and that they are mistreated, marginalized, and discriminated against because of their financial situation [24,26-28].

Thus, healthcare inequities persist even in publicly funded healthcare systems like those of Canada and the UK [29]. Healthcare access, which is responsive to a social determinant such as poverty, is influenced by many economic and social factors. PLPs often report that they only use health services that are low-cost or free and that they have unmet healthcare needs due to costs of transportation and medication [24-26]. Meanwhile, the scarce research conducted on HCPs’ attitudes toward poor patients and their perceptions regarding the economic and non-economic barriers encountered by PLPs in the healthcare system suggest HCPs usually have misconceptions about their disadvantaged patients’ situations that contribute to their negative attitudes toward them and stigmatization [22,30-32].

Few studies provide in-depth understanding of HCP’s responses to poverty as a risk factor for health and healthcare inequities. Even more scarce are studies exploring the perspectives of PLPs who are affected by HCPs’ non-responsiveness in a context of poverty. To address this gap, we developed EQUIhealThY, a participatory research project looking at primary care practices, in collaboration with ATD (All Together in Dignity) Fourth World, an international community organization devoted to supporting actions to eradicate poverty around the world. This study combined the perspectives of PLPs and healthcare teams to explore barriers to responsive care for deprived persons, with a view to fostering equity-focused interventions in primary care.

## Methods

### Research design

For this study, we used a participatory action research approach in which both academics and those for whom the research results are intended (i.e., persons living in poverty and healthcare teams) collaborated closely in all stages of the research [33-36]. This type of equitable participation usually allows the non-academic members to benefit immediately from the research findings and to become involved in the knowledge transfer process [37].

At the outset, we established a Steering Committee and a Monitoring Committee to promote the involvement of different groups of participants. The two principal investigators and two members of the ATD Fourth World International Volunteer Corps sat on both committees. The Steering Committee’s decision-making role included identifying the research question, developing data collection strategies, and participating in the data analysis, among others. It was made up of four members of ATD Fourth World (two PLPs and the two volunteers), two academic researchers (one of whom was also an HCP), one HCP, and two research assistants. Six members of the Steering Committee formed the Monitoring Committee, consisting of two academic researchers, two research assistants, and the two ATD Fourth World volunteers. This committee was responsible for the operational aspects of the study and had a decision-making role in planning, selecting methods and group facilitation approaches, and identifying training needs. It also played a role in data analysis (e.g. identification of key themes, descriptive analysis of merging of knowledge, etc.).

The methods used in this study were photovoice and the merging of knowledge and practice. Photovoice, often used in participatory action research, uses photography as a means of generating knowledge about the lived experience of participants [38-40]. Photovoice was first introduced in the 1990s by Wang and Burris [41], who used it with women in China’s Yunnan Province. Since then, it has been used with various populations, particularly to promote empowerment and critical consciousness among vulnerable groups [39,42-44]. According to Wang and Burris [41], there are three goals associated with photovoice: (1) to allow people to reflect on their community; (2) to provide, through photographs, the space to develop a critical dialogue and thinking about specific issues of concern; and (3) to generate concrete changes by reaching policy-makers.

The merging of knowledge and practice is a method developed by ATD Fourth World Movement, our main non-academic partner in this study. This method, which has been tested in Europe in similar initiatives, encourages the sharing of different perspectives by creating the necessary conditions for exchange and dialogue among three sources of knowledge: academic and theoretical knowledge (academic co-researchers), action and engagement knowledge (professional co-researchers), and existential and experiential knowledge (PLP co-researchers) [45]. The main focus of this method is to support the co-researchers’ continuous reflection and knowledge development in groups of their own peers and then to share this knowledge among peer groups to produce a merged result. Working with peers helps avoid inequalities in dialogue, as the PLPs’ full participation is not inhibited by unequal power relationships.

### Participants and sampling

This study was carried out in an urban community organization, ATD Fourth World, and two teaching family medicine practices. One practice is in a large urban metropolitan area; its patients include people on social assistance, people with substance dependencies, and immigrants living in poverty. The second is located in a semi-urban setting far from the city centre and serves poor workers, people on social assistance, and some first-generation immigrants.

Three photovoice groups were created, composed of participants from these three settings. The first group was comprised of six members of ATD Fourth World, of whom four were PLPs and two were the volunteers. The second and the third groups were made up of a total of 15 HCPs from the family medicine practices and included physicians, residents in family medicine, nurses, social workers, psychologists, and receptionists. The members of all three groups were trained in photovoice and participated in the merging of knowledge and practice meetings. All participants signed informed consent forms before participating in the study. The Charles-LeMoyne Hospital Research Centre Ethics Committee gave human subjects research approval for this project.

### Data collection and analysis

Each participant was invited to take photographs over a period of four weeks that responded to the Steering Committee’s research question: ‘What are the barriers between PLPs and HCPs?’ Subsequently, the participants met in their respective peer groups, with a trained facilitator, and presented their photographs. During these 2- to 4-hour meetings, participants shared their thoughts about the photographs and chose which photographs and reflections to share with the other groups. Next, ATD Fourth World members met with each HCP group in a half-day merging of knowledge and practice meeting. Sharing perspectives helped participants to better understand each other and to build common reflections. Both merging of knowledge and practice meetings were digitally recorded and then transcribed verbatim.

Data analysis was carried out in three steps. First, two research assistants developed a coding grid that was subsequently evaluated and improved by the Steering Committee. Once the final version of the grid had been accepted by all members of the Steering Committee, the research assistants coded the transcripts using NVivo 9. Finally, the research assistants prepared a summary of the analyzed data highlighting the themes raised, as well as the differences and similarities between the HCP and PLP groups. Three main themes were identified: the living conditions of PLPs, the organization and functioning of the healthcare system, and the quality of interactions between HCPs and PLPs. The first two of those themes were analyzed by two research assistants and two researchers, and interpretations were verified by the Steering Committee. For the theme of quality of interactions between HCPs and PLPs, the analysis and writing were done together over six months with all members of the Steering Committee. We first worked in subgroups for seven meetings, then in six meetings with the full committee, where we integrated the work of the subgroups. Approximately 60 group sessions were conducted between January 2012 and November 2013.

### Trustworthiness

Data triangulation, peer review, and an audit trail were used to ensure the rigour and quality of the findings. We triangulated a variety of data sources, including transcripts from meetings of different participant groups, different methods of data collection, researchers’ reflective journals, and newsletters addressed to co-researchers about the progress of the study. Peer review was achieved through regular phone meetings with experienced participatory researchers and a research facilitator, and through regular face-to-face meetings between academic and non-academic researchers.

## Results

We identified three main barriers that impede care responsiveness in a context of poverty: the living conditions of PLPs, the quality of interactions between HCPs and PLPs, and the organization and functioning of the healthcare system.

### Barriers pertaining to living conditions and medical needs

The precarious economic conditions of PLPs negatively impacted their lives. Participants identified three types of barriers related to living conditions: lack of financial resources, poor housing and environmental stress, and healthcare teams’ powerlessness.

#### Lack of financial resources

The inadequacy of the benefits provided by Quebec’s Social Assistance Program was raised several times. PLPs explained that it is impossible to maintain good health when living on social assistance, which, as of January 2013, provided $604 for a person living alone. This amount represents about half of the Market Basket Measure and is below the Low Income Cut-off in Canada. Having a job does not necessarily solve the problem, as minimum wage workers are also unable to escape poverty. PLPs must constantly fight to survive, paying close attention to their budget and struggling to meet their basic daily needs (e.g. food, commodities, clothing, transportation, etc.).

Illustrative of this, the topic of food arose frequently in relation to lack of resources. As one PLP participant explained, poverty severely compromises efforts to eat a proper diet:

*If you don’t have enough money, you can’t eat your fill. If you don’t have money, you get sick faster because you can’t buy the necessities. The doctor may well tell you what to eat, but if you don’t have the means to buy the basics to feed yourself properly, it doesn’t work.*

PLP participants also said that, despite knowing the basics of a good nutrition, it was impossible to achieve due to their financial situation. One gave the example of a diabetic patient who must calculate calories and follow a dietician’s recommendations while trying to stay within a tight budget. PLP participants also stressed the incompatibility of certain HCPs’ recommendations with their reality:

*The issue is the poverty itself, because when you live in a precarious situation you can’t take care of your health.... We seem irresponsible, but we can’t eat three meals a day. That’s why we’re not healthy. Then it affects our mental health, too.*

Thus, some HCPs’ demands are unrealistic for PLPs, considering their living conditions and financial situation. PLPs’ financial situation also has an impact on their access to healthcare. As one PLP participant noted:

*I waited 20 hours [in the emergency room], I went to two clinics, they told me… we can’t take you, there’s no room. The second clinic said… you can come back tonight. I said I didn’t want to run around, I was sick, I was very sick, I was sick as a dog. Was she going to pay for the bus? I’d had bronchitis since October, when I went to the emergency room. I waited 20 hours and I needed to eat, but I had no money, I had almost none.*

Long wait times or needing to go to more than one health clinic can generate additional expenses (e.g. transportation, babysitting, etc.). Moreover, the working poor may have work conditions that do not allow them the extra time needed (e.g. strict work hours).

#### Poor housing and environmental stress

PLPs live in unhealthy physical conditions that may seriously impair their physical and mental health. One PLP participant noted that it is difficult to be healthy without comprehensive care and that life expectancy in poor neighbourhoods is 10 years shorter than in wealthy neighbourhoods:

*…this is due to the stress of everyday life and to the means used to counter this stress that are harmful to health. For example, alcohol, cigarettes, and soft drugs. It’s easier to take care of your health if you’re rich. For example, if you’re depressed and you have the means, you can go rest in Florida. If you live in poverty, you return to a dump; it’s not the same thing.*

Finally, beyond their financial situation, PLP participants noted that HCPs do not always take into account living conditions such as stress or violence and, as such, have a poor understanding of PLPs’ daily reality. Overall, participants thought that unhealthy living conditions predisposed PLPs to develop chronic illnesses, mental health problems, various addictions, and a sense of isolation.

#### Healthcare teams’ powerlessness and lack of understanding of poverty

HCPs noted that people without a social network may lack support, especially in terms of physical and psychological health. PLP participants also confirmed that there is a link between poverty and isolation: “*It’s true that when we become poor … our social circle often gets smaller.”* Because of their isolation, PLPs are not always aware of the resources available to assist them in the healthcare system. Also, some HCPs said they felt powerless and poorly equipped to deal with the difficult life conditions and complex needs of PLPs:

*…sometimes you feel like an insignificant pawn, when it [the PLPs’ complex situation] is so big, big, big, big, and then I wonder whether we aren’t left too much to our own devices, we doctors? You know, sometimes we’re seen as all-powerful gods, but I think we should get down from our pedestal and say, well, we need some help, too, to manage a complicated situation like this.*

Indeed, some PLPs’ situations are very complex and require a lot of involvement on the part of HCPs, especially in terms of time and paperwork (filling out forms for work disability, social assistance, legal reports for jobs and housing, etc.). One physician gave the example of one of her patients to illustrate this situation:

*She was in her forties, she had hearing and visual problems, but it was all a bit too mild to be taken seriously. She was always living on the edge. Most of all, she was always excluded from employment and housing.... So now, nine years later, she’s working.... But it took nine years and an enormous amount of paperwork that I can’t always do if I only have 15 minutes to see a patient. That’s what I’m expected to do: see patients faster and see more of them.*

HCP’s sense of powerlessness is accentuated by the lack of resources to assist patients, especially when it comes to psychotherapy and physiotherapy, which are difficult to access within the free public healthcare system in Quebec due to long wait times, and which are costly in the private system. Thus, HCPs do not always feel able to provide the necessary care or optimal treatment:

*So there we are with our patient, and we feel overwhelmed and without resources; we can’t prescribe physiotherapy because the patient has no money. So, yes, the only way is often a prescription, because medication is more accessible, and we’re very aware this isn’t the optimal treatment, but we try to do what we can.*

One HCP participant noted that, while PLPs are sometimes aware of the resources available to them, particularly in community organizations, some obstacles (e.g. mental health problems, being disorganized, feelings of shame) prevent them from using them. Indeed, PLP participants said they had difficulty using certain resources, such as food banks, because they were embarrassed and felt too ashamed.

Finally, some HCP participants noted that there are not enough resources for helping PLPs, since poverty is not a medical condition, as is, for instance, substance abuse. As one HCP participant said: *For something to be recognized as serious, it has to be a disease. That’s what’s bad.* PLP participants reacted strongly to these comments, saying:

*Poverty isn’t a disease, but after a while, it becomes one, like it or not. Somebody who lives, I don’t know, five years in poverty.... I have a neighbour who’s very sick and he doesn’t want to be treated. He’s been living in extreme poverty for a very a long time. He wonders, if he recovers, whether he should keep on living or not. If he recovers from his illness, he’ll still be living like this. Is it worth it?*

### Barriers pertaining to the quality of interactions between HCP and PLP

Participants identified two types of barriers to good relationships between HCPs and PLPs: difficult communication and social distance. These barriers are intimately connected to living conditions, since HCPs often misunderstand the realities of PLPs.

#### Difficult communication

Both PLP and HCP participants noted that communication is often difficult between them, which negatively affects their relationship. From the PLPs’ perspective, physicians receive insufficient training in interpersonal skills and consequently cannot communicate effectively with them. One PLP illustrated physicians’ coldness with a photograph of a block of ice. However, HCP participants (mostly the family physicians and residents in family medicine) were surprised PLPs felt this way, because training in interpersonal skills had recently been added to the medical curriculum. A family physician participant stated:

*It was hard for us to hear that we, as a profession overall…were like blocks of ice.... There are a lot of expectations in terms of communication, so we work very hard. And, in the end, it has to be said that the people training physicians are aware of this issue, but we still don’t see the effects. We’d like to believe that this might be coming, more humane doctors.*

Communication difficulties between PLPs and HCPs may also be associated with their different levels of education. HCPs often use medical language without verifying whether their patients understand. PLPs’ lack of understanding may complicate the care relationship. One PLP participant pointed to lack of trust, fueled by past negative experiences, as another reason why communication is difficult. Finally, the shame felt by some PLPs can be so strong that they prefer to remain silent rather than to talk about their situation with HCPs.

#### Social distance

PLPs and HCPs live in different social contexts and do not face the same realities. These differences create *social distance*, which manifests as: 1) prejudice and labels; 2) different choices and logics, but similar values; and 3) social inequalities and unequal rights.

1. Prejudice and labels

All participants agreed that HCPs may hold prejudices toward PLPs. In this regard, one PLP participant said: *There is the barrier of the ‘welfare’ label. ‘Poor women’ equals stupid and lazy. There are prejudices that are a real barrier between PLPs and HCPs.* PLPs also thought that professionals held them to be responsible for their own health problems, and said this was because HCPs were not aware of their circumstances. Of all categories of HCPs, the PLPs identified physicians’ prejudices as the most harmful, as diagnosing from a position of prejudice could create much suffering.

The HCP participants also recognized that professionals sometimes held prejudices toward PLPs, attributing these prejudices to their own limitations, either in terms of their personal boundaries (e.g. they feel uncomfortable dealing with PLPs) or of the system (e.g. they are ill-equipped to deal with PLPs’ complex situations). Although HCP participants recognized that certain professionals may hold prejudices, few admitted to having negative preconceptions about PLPs themselves.

2) Different choices and logics, but similar values

In the merging of knowledge and practice meetings, it emerged that the PLP and HCP participants held the same values, despite their different choices founded on different logics. A striking example of the contrast in their two logics was their discussion about new mothers’ choice between breastfeeding or using infant formula. HCPs noted that PLPs would rather use formula than breastfeed, even though, in the HCPs’ opinion, breast milk is the best choice for babies’ health and also more economical. One HCP expressed her view of the behaviour of mothers living in poverty who choose not to breastfeed and instead spend money on formula, which is very expensive:

*As I see it, breastfeeding is easy, the milk is always warm and available, and it allows the baby to be very close to his mother, to establish a good contact. Several experts say breast milk is good, it has antibodies, lots of things; and then when patients tell me they’re not going to breastfeed, and they’ll buy formula, well, I see that as a barrier. Especially for someone living in poverty, I think it's a shame because we see the price here on the sign, formula is very expensive, whereas breast milk is free.*

In contrast, a PLP participant explained that the choice to use formula may be based on the fear of not being able to give babies everything they need in breast milk. Mothers may believe their milk is not good enough because they were often told they were bad and a failure, so they devalue themselves:

*Yes, because the choice she makes is: If I’m not eating enough, will my milk be good? Will it have everything it needs? It’s very doubtful, when you eat once a day, and then you eat bread and butter, no matter the menu, it’s not much. You know? You say to yourself that formula, even at $20, at least it’ll have everything [the baby] needs.*

Hence, the choices are based on different priorities, which lead to misunderstanding and judgment on both sides. As one HCP participant said:

*I couldn’t possibly have known that in the office. I would have asked the woman whether she’s breastfeeding or giving formula. She’d say she’s giving formula. I’m not sure I’d ask her why, and I’m also not sure that it would come out, ‘Well, I give formula because I’m afraid my child won’t get everything he needs in my breast milk, because I don’t eat enough.’ Maybe she’d never tell me she was eating only once a day and that this made her worried about her baby. So I go out of there thinking she’s buying formula, which is super expensive, and that’s not a good choice. And she has the impression I don’t understand her.*

Thus, the different choices are based on different logics, as one PLP participant noted:

*It’s sad, because everyone wants to be a good mother. But for this woman, being a good mother means breastfeeding. And for another woman, being a good mother means feeding your baby formula. So we have the same values: we want to be good mothers to our children.*

Overall, PLPs and HCPs, given their different backgrounds and life experiences, have different logics. PLPs are often trapped in the ‘*logic of no choice’*, seeing themselves confined to only one solution because of their life conditions (e.g. low income, marital status, unaffordable housing), while perceiving that HCPs have strong opinions about what choices they should make. As one PLP participant noted:

*To realize that your opinion of someone who has more money is different from [your opinion of] someone with less; [the former] has a choice. When something is imposed, you have no choice. You have just one option. And that’s what you have to do, because if you don’t, you’re not a good poor person, you don’t do the right things, you’ve missed the boat a little’. That irritates me!*

In addition, when PLPs make a choice in their daily lives, they often feel judged. One PLP participant said: *Do we have the right to buy a beer without being judged? Without being judged by society? Are we allowed to have fun, to make choices?*

In this ‘*logic of no choice’*, PLPs feel guilty when they are not able to follow the recommendations of HCPs (e.g. regarding nutrition, breastfeeding, etc.). They feel they have the know-how and the intelligence to follow them, but their living conditions are major obstacles that may lead them to make different choices.

Our participants discovered they lived under a ‘*logic of no choice’* and realized that HCPs’ actions towards them were guided by a *‘logic of diagnosis’*. According to all participants, the *‘logic of diagnosis’* is central to HCPs’ work. With limited resources and time, physicians may reach a diagnosis before completely understanding the patients, including their psychosocial situation. They feel this is the way things should be done, even though they accept that this approach might not work for all patients. As one HCP participant noted:

*You know, it takes a diagnosis to treat a patient. And maybe there’s a flaw there and it doesn’t work for all types of people. Then there’s also the fact that we’re expected, with our limited resources, to analyze this patient in half an hour and decide what we’re going to do with him, because the next time we see him may be in six months.*

PLPs felt that this situation could have serious consequences, because without taking the time to see the whole situation the HCP could be making a diagnosis based on prejudice. One PLP participant reported that a friend was misdiagnosed with intellectual disability after one consultation of only 15 to 20 minutes:

*The physician makes an instantaneous reading, like a Polaroid. You wait a couple of seconds, and then you have the picture. He doesn’t see the overall situation, the stress or the violence you live with. It’s a fragmented vision. Then we’re locked into this diagnosis that the social workers and other health professionals rely on. It follows us our whole life.*

Participants spoke about the need to find a meeting point between the contrasting logics of these different worlds. For example, an HCP participant explained her photograph:

*What this represented for me, is also the gaps between what we expect of the patient—you’ve talked about it a little: the food guide, what should be done, what’s good—we know, but we can’t do it with what the patient can afford. So what we need to do, in teaching, is to consider people’s purchasing power, so that we can meet in the middle and work together.*

However, one PLP challenged the HCP by saying that, although it is a good thing to want to ensure these two different worlds meet, the power imbalance makes it difficult:

*Individually, PLPs have very little power over their own lives, but those who try to improve the social situation, it must be said, are totally excluded, they’re not listened to by governments. Those of you who live in the other world as doctors or who rub shoulders with people in power, what can you do…?*

3) Social inequalities and unequal rights

Some participants recognized that PLPs have little social, economic, and political power and social recognition compared to HCPs, who typically come from a higher socioeconomic class. PLP participants were aware of their position and wished that HCPs would offer more help and support. Conversely, HCPs (especially family physicians) thought they were too often perceived as having more power than they actually have. As one noted:

*We’re ready to work to change things, but at this point we’re in the same position. We don’t know the ropes, we don’t know where to go…. I’m only a physician, and I’m not pretending to be anything else. So I don’t have any more pull to play the system, I don’t have any more pull to be heard. What I say is not going to be heard any more than what you say.*

Some HCP participants initially had the impression that PLPs were accusing them of being the cause of their problems. Then, during the discussion, both groups came to agree that it is the system that produces the barriers and perpetuates social inequality. One HCP participant concluded:

*I think the communication between PLPs and physicians is probably not so bad. Maybe it’s the entire system, and everything around it, that really needs to be reviewed in depth.*

In the end though, participants agreed that, while the system is partly responsible for some of the barriers, everybody has an individual responsibility to break them down.

### Barriers pertaining to the organization and functioning of the health care system

PLP and HCP participants discussed the organization and functioning of the Quebec healthcare system, which tends to create and reinforce barriers. Three categories of barriers were identified: lack of resources, difficult access to care, and healthcare system complexity.

#### Lack of resources: shortage of medical staff and limited time

The lack of equity-focused interventions in the healthcare system results in staff shortages and, consequently, overall low quality of care. HCPs noted that they are not equipped to meet the needs of PLPs and may feel helpless. They described feeling pressured to perform well at the least cost to the system, which necessarily has implications for the time and services they can provide to the patients. As one HCP participant noted:

*If you only knew the pressure! We have nightmares when we hospitalize people, because we can get three or four calls a day asking us to discharge someone who has no reason to remain there. But sometimes it’s against our values to discharge the patient because he has nowhere else to go. But the pressure is really strong.*

The lack of time limits HCPs’ ability to fully understand their patients’ situations, to convey all necessary information while ensuring patients’ understanding, to make accurate clinical assessments, to complete all administrative work effectively (e.g. filling out government forms), and to carry out preventive interventions. Moreover, taking more time with a patient in need delays their schedule, causing frustration in other patients. HCPs suggested that improving the multidisciplinary functioning of the healthcare team could help solve these problems.

From the PLPs’ perspective, lack of time during HCP consultations generated stress. They said they felt pressed to the point that they sometimes forgot to mention important issues and feared the time constraints would affect the HCPs’ clinical evaluation, with the attendant potential for misdiagnosis.

#### Difficult access to care

All participants recognized that limited healthcare system resources made access to care difficult. For HCPs, the most significant problem was the shortage of family physicians in the province, which negatively impacts patients’ health. They pointed out, for example, that patients often go to emergency rooms after failing to get appointments at primary care clinics as their untreated conditions worsened. Additionally, access to specialized care (e.g. physiotherapy, psychotherapy, nutrition) is problematic for PLPs because they have to consult in the public healthcare system, where wait times, according to our participants, are over a year. Indeed, HCP participants indicated that private medical insurance guarantees better access to care and faster recovery. Given their awareness of the negative consequences of wait times on patients’ conditions, HCPs expressed frustration in dealing with patients who do not have private insurance. As one HCP participant said:

*The first question we ask is: ‘Do you have insurance?’ If the person says no, we’re sorry, because we know we’ll refer them to the public system, but it will take months. A person with a higher income would go to the private, and bing, bang, it will happen right away.*

PLP participants also highlighted the difficulties of trying to get in touch with their family physician by telephone. Generally, they must first contact the secretary or nurse who, as intermediaries, may inadvertently distort messages.

#### Complexity of the healthcare system

Our participants considered healthcare system complexity to be an important barrier. The system, with its bureaucracy and administrative and legal regulations, offers rigid, standardized services and fails to meet the specific needs of PLP patients. Also, according to the HCP participants, it is difficult for patients to navigate the healthcare system and to understand the processes and procedures required. It can be difficult for patients to know where to consult for certain health problems (e.g. whether to make an appointment at a primary care clinic or go to the emergency room). It can also be difficult for patients to find their way to various departments in large healthcare facilities. HCPs suggested that different departments should be grouped together to eliminate confusion.

Participants also raised the issue of social assistance benefits. In Quebec, determining patients’ social assistance needs is under the purview of physicians. It is they who declare a patient fit or unfit for work, which influences the amount of benefits granted by the government. A person who is declared unfit for work receives $314 more per month than one who lives alone and is declared fit for work (effective January 1, 2013). One PLP participant said:

*[For us] to get 900 [dollars] per month, physicians need to fill out forms. They don’t always want to do it because they’re afraid of being contested by the Social Assistance Program. The barrier is that the law requires physicians to decide whether you’ll have 600 or 900 [dollars] a month to live on.*

PLPs who believe they are eligible for the additional disability amount need physicians to complete specific paperwork, but physicians may feel uncomfortable performing this task. Physicians find themselves in a dilemma because they are aware of the impact their decision has on the lives of PLPs. For our participants, this government policy has created inequities and stress for persons receiving social assistance as well as for doctors, who want better life conditions for their patients and a good care relationship. As expressed by a physician:

*Just to say, I agree it’s pretty hellish. We’re filling out forms and entering things just to enter things. And even if I put those things in, it works for a while and then one day they [bureaucrats with the Social Assistance Program] don’t buy it anymore.... It worked, and suddenly it’s not working anymore, the check is cut, the person doesn’t even know. So we feel very badly, too.*

## Discussion

This study is among the few to explore perceived barriers to responsive healthcare for PLPs and to combine their perspective with that of HCPs on this important issue. Our study revealed that unhealthy living conditions prevent PLPs from maintaining good health and accessing care, with a negative impact on their interactions with HCPs. One major barrier to appropriate care for PLPs is HCPs’ lack of knowledge and understanding of these unhealthy conditions and of their impact on the health of PLPs. Our participants also criticized the healthcare system for not providing adequate resources to address the complex needs of PLPs. These barriers exist despite the fact that Quebec has a publicly funded healthcare system that is mostly free.

Before interpreting our results, we should point out the limitations of this study. First, it reflects the experience of only a limited number of participants, although we consider this number to be satisfactory for a participatory research design. Indeed, limiting the number of participants is usual practice in participatory research, as it fosters active participation of the vulnerable non-academic researchers [46]. Participatory research is time-consuming, and effort was required to sustain PLPs’ participation and avoid their instrumentalization during the research process [47]. Promoting their participation required training, guidance, support, and time to prepare them and help them to feel free to express their views in the presence of health professionals. We did not recruit PLPs who were patients of the HCPs participating in this study. PLP participants were helped and supported by one volunteer from ATD Fourth World, who met with them before each meeting with healthcare teams and/or researchers. Nonetheless, asymmetric or hierarchical relationships between nurses and doctors or between receptionists and doctors and/or between PLPs and researchers may have influenced the data we obtained.

Our results suggest that healthcare system complexity and the lack of resources, at both the system and personal levels, are major barriers in providing responsive care to PLPs. Indeed, it is known that poverty is associated with various sociocultural barriers to access to care [15-18,32,48]. Some of these are related to the characteristics of PLPs, such as culture, gender, or health literacy level [49]; others are sociocultural or economic and pertain to the healthcare system. For instance, costs related to healthcare have been increasing for several years in Quebec and affect the use of health services by those in low-income households [50].

Moreover, a recent report from the Canadian Medical Association noted that many obstacles in the healthcare system prevent HCPs from providing equity-focused care [51]. Weak integration of health services, deficient physician remuneration systems, and HCP unsympathetic attitudes and powerlessness toward patients’ low socioeconomic status were among the top items in the list of obstacles [51]. Substantial changes must be implemented to better address the needs of deprived persons in the healthcare system, as reported by other studies [13,23,25,48]. Among these changes, we suggest that compensation models for health equity practices should be tested, implemented, and evaluated for effectiveness before being scaled up. We also suggest allocating more resources to support interdisciplinary healthcare teams in underserved areas and to modify primary care practice guidelines to incorporate social and economic factors.

Some studies have reported that some HCPs are aware of the barriers faced by PLPs in the healthcare system [20,47,52]. For instance, Bloch et al. [20] found that HCPs with academic and/or frontline expertise in poverty agreed that PLPs face “numerous structural, attitudinal, and knowledge-based barriers”, including lack of access to transportation, lack of insurance, “limited help-seeking linked to stigma and shame at personal circumstances, inflexible practice rules that are difficult for PLPs to comply with, billing structures that discourage longer appointments, unwelcoming practice environments, and [HCPs’] lack of familiarity with the social security system and relevant community-based resources.” Participants in our study also recognized that, generally, HCPs do not have a substantive understanding of PLPs’ lived reality and that accessing patients’ socioeconomic information is often difficult, which may lead to prejudicial attitudes and feelings of being overwhelmed and powerless. Our results corroborated those of others regarding the need to develop effective training that integrates evidence on the social inequalities of health and poverty to provide the knowledge and tools required to help healthcare professionals avoid stigmatizing and developing negative attitudes towards the poor [20,24,25,52].

Previous studies have underscored the need to close the equity gap in healthcare systems by better addressing the social determinants of health, such as poverty, poor housing, and food insecurity. Yet an important finding of this study, shared by all participants, is the need to alter the law that mandates physicians to determine whether or not patients are fit for work, hence determining the amount of social assistance they receive and consequently the living conditions that impact their health and healthcare. The current minimum wage in Quebec is not sufficient to maintain good health or to deal with the costs of self-care, including healthcare itself, given that the public system in Quebec is increasingly under pressure of privatization. First, low wages result in inadequate housing, food insecurity, and sometimes avoidance of healthcare because of transportation costs [24,25,53]. Second, this study has important implications for patient–provider interactions. Barriers faced by PLPs intersect with HCPs’ sense of powerlessness and lack of resources, which affects the quality of communication, something which is crucial for creating space for dialogue, trust, and mutual engagement.

## Conclusions

Our research revealed that unhealthy living conditions prevent PLPs from accessing quality healthcare and maintaining good health. Also, the complexity of the healthcare system’s organization and functioning has a negative impact on PLPs’ interactions with healthcare providers. Changes in policy and practice are needed to address those barriers and to achieve greater equity and provide more responsive care for persons living in poverty.
